# Immune anticipation of mating in *Drosophila*: *Turandot M* promotes immunity against sexually transmitted fungal infections

**DOI:** 10.1098/rspb.2013.2018

**Published:** 2013-12-22

**Authors:** Weihao Zhong, Colin D. McClure, Cara R. Evans, David T. Mlynski, Elina Immonen, Michael G. Ritchie, Nicholas K. Priest

**Affiliations:** 1 Department of Biology and Biochemistry, University of Bath, Bath BA2 7SW, UK; 2 Department of Ecology and Genetics, Animal Ecology, Evolutionary Biology Centre, Uppsala University, Norbyvägen 18 D, Uppsala 75236, Sweden; 3 School of Biology, Biomedical Sciences Research Complex, University of St Andrews, St Andrews, Fife KY16 9ST, UK

**Keywords:** immune anticipation, sexually transmitted infections, ecological immunology, *Drosophila melanogaster*, *Metarhizium robertsii*, innate immunity

## Abstract

Although it is well known that mating increases the risk of infection, we do not know how females mitigate the fitness costs of sexually transmitted infections (STIs). It has recently been shown that female fruitflies, *Drosophila melanogaster*, specifically upregulate two members of the *Turandot* family of immune and stress response genes, *Turandot M* and *Turandot C* (*TotM* and *TotC*), when they hear male courtship song. Here, we use the *Gal4/UAS* RNAi gene knockdown system to test whether the expression of these genes provides fitness benefits for females infected with the entomopathogenic fungus, *Metarhizium robertsii* under sexual transmission. As a control, we also examined the immunity conferred by *Dorsal-related immunity factor* (*Dif*), a central component of the *Toll* signalling pathway thought to provide immunity against fungal infections. We show that *TotM*, but not *TotC* or *Dif*, provides survival benefits to females following STIs, but not after direct topical infections. We also show that though the expression of *TotM* provides fecundity benefits for healthy females, it comes at a cost to their survival, which helps to explain why *TotM* is not constitutively expressed. Together, these results show that the anticipatory expression of *TotM* promotes specific immunity against fungal STIs and suggest that immune anticipation is more common than currently appreciated.

## 1. Introduction

Mating is fraught with danger. In addition to the fitness costs associated with finding sexual partners, copulation and offspring production, mating increases the risk of acquiring sexually transmitted infections (STIs) [1–3]. In insects, STIs are often both highly prevalent and pathogenic [3,4]. It is generally thought that they exert a selective pressure strong enough to influence the evolution of mating systems, life histories, sexual conflict and sexual behaviour [3,5]. Yet, we have a poor understanding of how they have shaped the immune system [6].

Females could mitigate the risks of acquiring STIs through immune anticipation of mating, the activation of immune responses before sexual congress and potential exposure to pathogens [7]. Pre-emptive immune activation is predicted to be more advantageous than a purely reactive response because it shortens the time delay of the immune response, and thereby maximizes its efficiency ([7]; MT Siva-Jothy, E Harney, W Zhong 2013, unpublished data). We know that females upregulate a number of immunity-related genes in response to mating [8–12]. But even the act of courtship might stimulate immune activation. If immune genes expressed during courtship represent immune anticipation of mating, then we would expect such responses to enhance immunity against STIs and to exhibit costs in some aspects of life history, because otherwise they would be constitutively expressed [13,14].

One way to address this possibility is to identify candidate immune genes associated with courtship and perform infection and fitness assays in which the expression levels of the genes are manipulated. Recently, *Turandot C* and *Turandot M* (*TotC* and *TotM*), members of the *Turandot* family of immune and stress response genes, were shown to be upregulated in the heads of female *Drosophila melanogaster* stimulated by male courtship songs independent of any physical encounter with males [15]. Of the two, *TotM* is probably the better candidate for anticipatory immunity against STIs, as it is poorly induced by non-immune-related stress [16] but strongly induced by both fungal infections [16–18] and mating [9,19,20]. In addition, induction of *TotM* by natural fungal infection exhibits similar fold-change in expression to well-known antifungal antimicrobial peptides (AMPs), including *Drosomycin* and *Metchnikowin* [18]. Surprisingly, there is little evidence that courtship stimulates the upregulation of the canonical *Toll* and *Imd* pathway immune genes, such as *Dorsal-related immunity factor* (*Dif*), an NF-**κ**B-like factor that regulates *Toll*-dependent immune responses thought to provide immunity specifically against Gram-positive bacteria and fungi [15,21,22].

Previous efforts in establishing *D. melanogaster* as a model laboratory system for studying insect STIs have focused on bacterial pathogens [23,24]. However, entomopathogenic fungi might be more appropriate. First, entomopathogenic fungi are widespread across diverse environments causing a large proportion of all known insect STIs, and indeed the majority of all insect diseases [3,25]. Second, because fungal spores cause infection through direct contact with the cuticle [26,27], they are amenable for comparisons between sexual and non-sexual horizontal transmission. Finally, studying the sexual transmission potential of entomopathogenic fungi in the laboratory have important implications for their application in the field as agents of biocontrol [28–30].

Here, we examine the hypothesis that *TotM* provides protection against sexually transmitted *Metarhizium robertsii*, a generalist soil-borne entomopathogenic fungus, which exhibits both sexual and non-sexual transmission in dipterans and has been used extensively in biocontrol [25,31,32]. Specifically, we test the predictions that (i) *Metarhizium* can be sexually transmitted in *D. melanogaster*; that (ii) expression of *TotM* helps to mitigate the cost of infections under sexual transmission, but not direct modes of transmission; and that (iii) the expression of *TotM* has fitness costs in the absence of sexually transmitted *Metarhizium*. To address these questions, we use the Gal4/UAS RNAi-targeted gene knockdown approach [33], in conjunction with large-scale demographic analysis, to estimate the immunity and fitness conferred by *TotM*, *TotC* and *Dif* under both STIs and high-dose direct topical infections (DTIs) of *M. robertsii*.

## 2. Material and methods

### (a) Fly strains and fungal culture maintenance

A wild-type Dahomey strain of *D. melanogaster* (provided by Dr Stuart Wigby, University of Oxford) was kept in large population cages (1 m^3^) with overlapping generations for 2 years prior to the start of the experiments. RNAi strains were obtained from Vienna *Drosophila* RNAi Center (UAS-*TotM*-IR, transformant ID 106727; UAS-*TotC*-IR, transformant ID 106379; UAS-*Dif*-IR, transformant ID 30579). We used the non-tissue-specific Act5C promoter to drive ubiquitous expression of Gal4 and UAS constructs (Act5C-Gal4/CyO, Bloomington Stock Center stock number 4414). We crossed Act5C-Gal4/CyO females with males carrying one of the UAS constructs to generate the active knockdown genotypes (Act5C-Gal4/UAS-*TotM*-IR; Act5C-Gal4/UAS-*TotC*-IR; Act5C-Gal4/UAS-*Dif*-IR). As a control for the presence of the UAS transgene, we crossed w1118 wild-type females (the genetic background for all RNAi lines, obtained from Bloomington Stock Center) with males carrying one of the UAS constructs (UAS-*TotM*-IR/+; UAS-*TotC*-IR/+; UAS-*Dif*-IR/+). As a control for the presence of the Gal4 driver, we crossed Act5C-Gal4/CyO females with w1118 males (Act5C-Gal4/+). The effectiveness of RNAi knockdowns of *TotM* and *TotC* was confirmed by semi-quantitative PCR [34]. All experimental animals were maintained at 25°C with 12 L : 12 D cycle in standard *Drosophila* vials at low densities (approx. 50 flies/vial) for at least two generations prior to the start of experiments. We used an oatmeal–molasses–agar media with added live baker's yeast and an antifungal agent (Nipagin), which inhibited the growth of naturally occurring saprophytic fungi. All experimental flies used were collected as virgins over a period of 24 h.

*Metarhizium robertsii* (isolate 2575, previously known as *Metarhizium anisopliae* strain ME1) was obtained from the Agricultural Research Service Collection of Entomopathogenic Fungal Cultures (ARSEF, United States Department of Agriculture). We inoculated quarter-strength sabouraud dextrose agar (SDA) with *M. robertsii* conidia (asexual fungal spores) and incubated the plates at 28°C for four weeks before storing at 4°C for up to three months. Conidia were collected by scraping the surface of the sporulating culture with an inoculating loop.

### (b) Sexual transmission of fungal pathogen

We assessed the transmission potential of *M. robertsii* by exposing healthy Dahomey females to males that had been topically inoculated with the fungus. At adult age day 4, groups of 10 virgin males were topically inoculated with 6 mg of conidia without CO_2_ anaesthesia by shaking in a 250 ml conical flask for 20 s. Inoculated flies were held in temporary holding vials for 24 h, ensuring that they had opportunities to groom themselves, which has previously been shown to be effective at removing fine dust particles [35]. At adult age day 5, each infected male fly was introduced into a new vial containing 10 uninfected virgin females of the same age and removed after 24 h. The logic of giving males time to groom and subsequently using a fresh vial was to allow male to adopt a more natural behaviour [32] and to minimize the probability of females contracting infection from conidia that had been dislodged during grooming. We then transferred and held treated females in individual vials for a further 24 h to allow egg-laying. The presence of larvae 4 days after oviposition indicated that the female had mated with an infected male. We assessed the infection status of females by the presence of *Metarhizium*-like fungal growth on cadavers. Flies were briefly immersed in 70% ethanol before being gently crushed and placed in Petri dishes on moistened filter paper at the end of the egg-laying period. After an incubation period of 5 days at 28°C, we examined all cadavers for signs of *Metarhizium*-like fungal growth (either hyphae or conidia) with a low-power dissection microscope. Because high levels of horizontal transmission of conidia between infected and naive files owing to non-sexual contact could confound our interpretation, we also assessed the potential for non-sexual horizontal transmission of *M. robertsii* using the same procedures described above by exposing naive males and females to infected flies of the same sex.

### (c) Survival assays under direct topical infection and sexually transmitted infection

We assessed the effects of gene knockdowns on survival under high-dose DTIs and sexual transmission (STI) using adult flies for all genotypes. For DTI, at adult age day 7, we infected groups of approximately 300 mixed-sex flies of each genotype with 20 mg of conidia, or kept as uninfected control, following the protocol described previously. Inoculated flies were held in temporary holding vials for 30 min before being transferred to demography cages (10 × 15 cm). For STI, we first inoculated 6-day-old w1118 males in groups of 20 with 12 mg of conidia, and then transferred 20 infected or control males with 20 uninfected females to demography cages at adult age day 7. As infected males in STI treatment suffered much greater mortalities than control males, we restored the original complement of 20 infected males by adding freshly infected w1118 males at day 12 and 24 postinoculation. For both DTI and STI, we removed and recorded dead flies daily until day 9 postinoculation and every 2 days thereafter. We also tracked the changes in pathogen loads in the first 24 h following DTI by sampling inoculated Dahomey wild-type flies at three time points postinoculation (0, 2.5 and 24 h; *n* = 9). Sampled flies were individually homogenized in 200 µl of 0.04% Tween80, diluted by a factor of 10^3^ and spread onto standard SDA plates. Pathogen loads were assessed by counting the numbers of colony forming units (CFUs) following incubation at 28°C for 24 h.

### (d) Fecundity assay under sexually transmitted infection

We assessed the effects of gene knockdowns on survival and fecundity of females exposed to fungus-infected males using flies from the same cohort collected for survival assays. In the fecundity assay, we first infected 2-day-old w1118 wild-type adult males (the genetic background of our RNAi strains). At 24 h-postinoculation, infected or uninfected control males were transferred to individual vials containing a single uninfected virgin female for each genotype. The mating pairs were assigned positions in randomized blocks and transferred to new vials after 24 h, and thereafter every 2 days until day 9 (*n* = 55/treatment/genotype). Used food vials were frozen 18 days after collection and the numbers of eclosed pupae were counted giving a combined measure of fecundity and larval viability. We assessed the proportion of females that became infected through mating with infected males by sampling all surviving females at the end of day 9 postinoculation (96.8%, 701/724) and checking for signs of *Metarhizium*-like fungal growth after incubation at 28°C for up to two months.

### (e) Statistical analysis

All statistical analyses were performed with R version 2.15 [36]. We assessed the contribution of mating to the transmission of STIs by comparing the proportions of flies that displayed *Metarhizium*-like fungal growth for mated females, and those that were kept with infected males but remained virgin using χ^2^-tests with continuity correction. We used student's *t*-test on CFUs to directly compare pathogen loads immediately after inoculation and after 24 h.

Cox proportional hazard regressions were used to analyse all survival data. The full model (including all genotypes) contained age at death and censoring information as the response variables—genotype, infection treatment and their interaction—were included as predictor variables. A separate Cox regression was performed for each gene of interest that only included the relevant knockdown and control genotypes (e.g. for *TotM*, the data included these genotypes: Act5C-Gal4/UAS-*TotM*-IR, Act5C-Gal4/+ and +/UAS-*TotM*-IR). For each gene of interest, we first extracted the hazard ratios (the fold-increase in risk of death in infected animals relative to uninfected controls) for the knockdown genotype and its combined control genotype (by pooling raw survival data of the relevant control genotypes) from the Cox models. Because the mortality rate in the DTI treatment is substantially higher than that in the STI treatment, it is difficult to directly compare the effect of immune gene knockdowns in the two treatments. To overcome this problem, we calculated normalized hazard ratios by dividing the hazard ratios of each knockdown by its associated combined control genotype. Unlike simple metrics of lifespan, this measure describes the effect of each gene knockdown on immunity after accounting for its genetic background, which allows us to directly compare the immune properties conferred by genes under STIs and DTIs, despite great differences in effect size. We assessed the survival cost of gene expression in the absence of infections by comparing the hazard ratios of each gene knockdown relative to its combined control genotype under uninfected control conditions.

We used mixed effects models to assess the effects of genotype and infection on fecundity across time. The full model included the number of eclosed pupae produced at each time point as the response variable; genotype, treatment, time and all associated two-way interactions as fixed effects (three-way interaction was non-significant when fitted, and thus dropped from the full model), and individual females as random effect (intercepts). We also included the age at death of male partners as a covariate in the full model to account for the possibility that females might have lower fecundity under STI simply owing to a lack of remating opportunities as infected males die at earlier ages than uninfected controls. Female fecundity in the first 24 h was excluded from the model as the fecundity was much lower than that at other time points and previous experiments suggested minimal *in vivo* fungal growth in this period (VL Hunt, W Zhong, CD McClure, DT Mlynski, EML Duxbury, AK Charnley, NK Priest 2013, unpublished data). We assessed the fecundity cost of gene expression in the absence of infections by comparing the mean total pupae productions of the gene knockdown (day 0–9 posttreatment) and the combined control genotype using one-way analysis of variance.

## 3. Results

### (a) Sexual transmission of fungal pathogen

**Figure 1.**
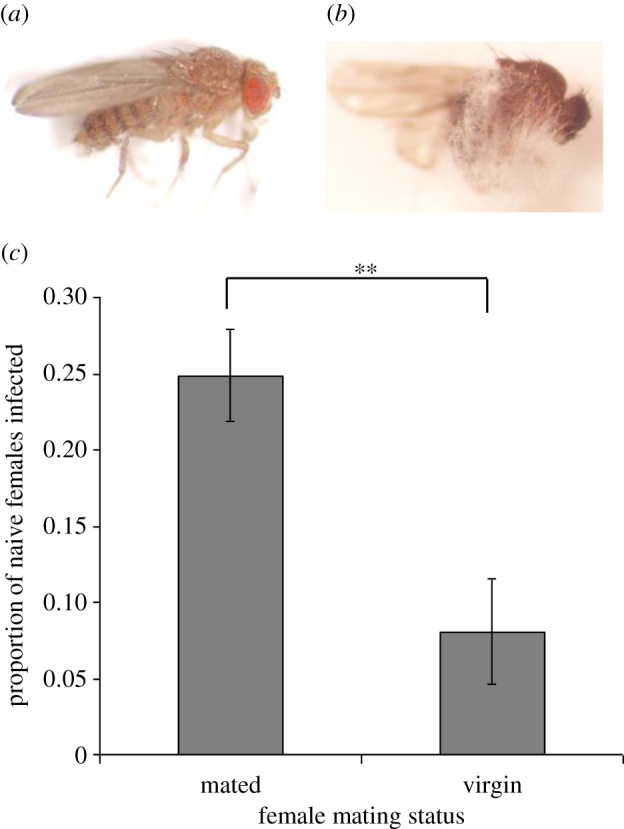
*Metarhizium robertsii* can be horizontally transmitted in *Drosophila melanogaster* as a result of mating. (*a*) A female *Drosophila* covered in *Metarhizium* conidia immediately after DTI, (*b*) growing hyphae of *Metarhizium* emerging from infected fly cadaver and (*c*) when kept in a cage with a *Metarhizium*-inoculated male, females that had been inseminated were much more likely to acquire conidia than those that remained infertile. (Online version in colour.)

We found that *M. robertsii* can be sexually transmitted in the fruitfly, with approximately one in five (55/263) naive females displaying *Metarhizium*-like fungal growth on their cadavers after being placed with a topically infected male for 24 h (figure 1*a,b*). Further analysis showed that fungal transmission was driven primarily by mating, as the proportion of cadavers with fungal growth was higher in gravid females than that infertile females (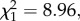 *p* = 0.0028; figure 1*c*). The dose received by females was likely to be low as the pathogen load of the topically infected males was only approximately 5000 CFU, which had declined by grooming from the initial load of approximately 20 000 CFUs (*t* = 7.69, *p* = 0.006; electronic supplementary material, figure S1). Finally, we also found that *Metarhizium* could be transmitted among same-sex flies (7/277 for male-to-male transmission and 7/266 for female-to-female transmission; electronic supplementary material, figure S2). Nevertheless, naive flies were much more likely to be infected through sexual transmission than through non-sexual transmission, 20.9 versus 2.6%, respectively.

### (b) Effects of sexually transmitted infection and direct topical infection on survival across RNAi strains

**Figure 2.**
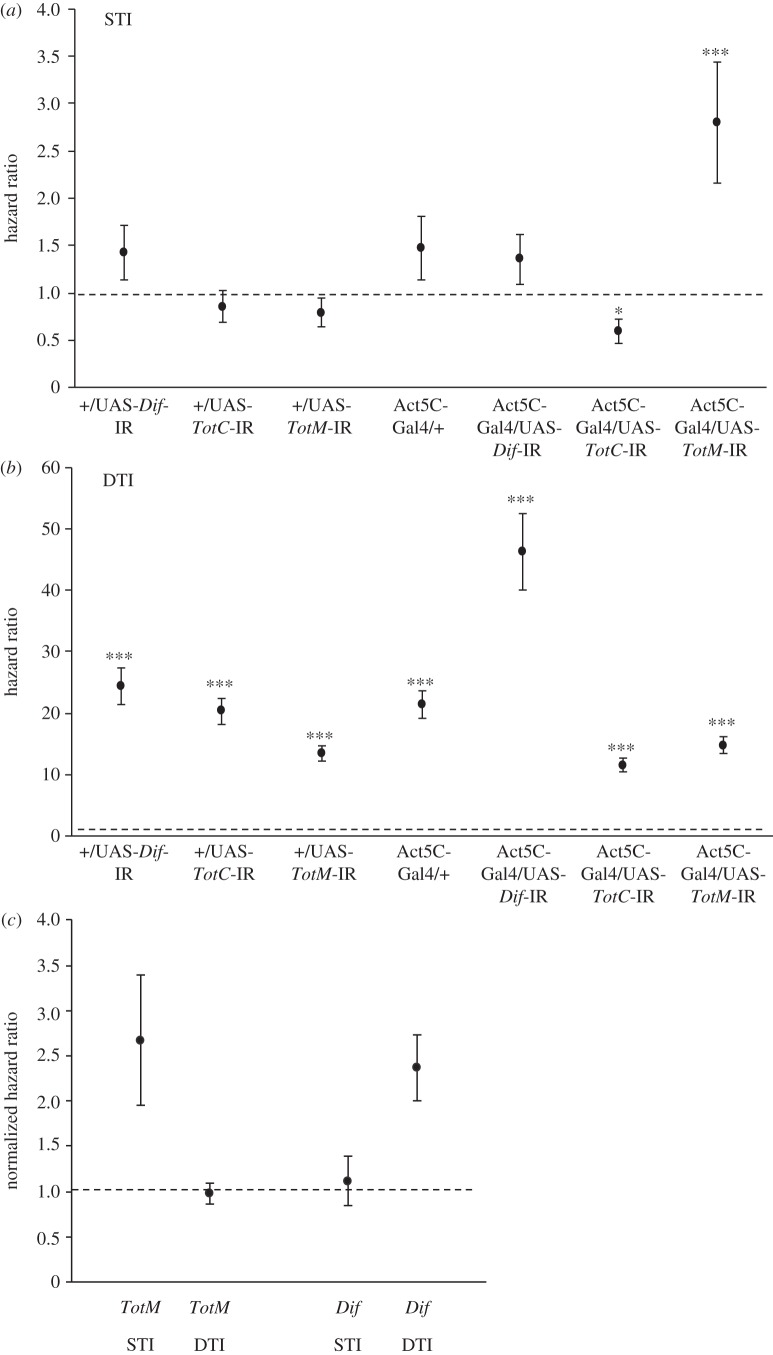
*TotM* is required for enhanced survival under STI, but not under DTI. (*a*) Cox proportional hazard ratios of STI relative to uninfected controls, (*b*) Cox proportional hazard ratios of DTI relative to uninfected controls and (*c*) the susceptibility of *TotM* and *Dif* under both STI and DTI after normalization for differences in the influence of mode of infection on hazard of the control genotypes. Dotted lines indicate hazard ratio of 1, which indicate both infected and uninfected controls had the same risk of death. Asterisks (*) indicate the level of statistical significance of hazard ratios (*p < 0.05; **p < 0.01; ***p < 0.001).

We found that *TotM* promotes immunity against *Metarhizium* when it is sexually transmitted (STI), but not when it is applied as a DTI. The effect of STIs on the hazard ratio, which estimates the risk of death in infected treatments relative to control treatments, was highly dependent on the host genotype (overall: genotype × treatment, 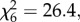 *p* = 0.0002; figure 2*a*). Specifically, *TotM* knockdown flies (Act5C-Gal4/UAS-*TotM*-IR) were susceptible to STIs, but there was no evidence of susceptibility in either of +/Act5C-Gal4 or +/UAS-*TotM*-IR control genotypes (genotype × treatment; 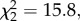 *p* = 0.00037; figure 2*a*). By contrast, there was no difference in susceptibility to STIs among *Dif* knockdown flies (Act5C-Gal4/UAS-*Dif*-IR) and its associated control genotypes +/Act5C-Gal4 and +/UAS-*Dif*-IR (genotype × treatment, 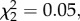 *p* = 0.98). Surprisingly, *TotC* knockdown flies (Act5C-Gal4/UAS-*TotC*-IR) had slightly higher survival postexposure than both of control +/Act5C-Gal4 and +/UAS-*TotC*-IR genotype flies (genotype × treatment, 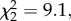 *p* = 0.011; figure 2*a*).

We found different patterns under DTI. While DTIs generally caused very rapid mortalities such that 95% of flies died within 9 days, some genotypes were much more susceptible (overall: genotype × treatment, 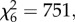 *p* < 0.0001; figure 2*b*). As expected [22], *Dif* knockdown (Act5C-Gal4/UAS-*Dif*-IR) females were significantly more susceptible to DTIs than either of its control genotypes (genotype × treatment, 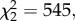 *p* < 0.0001; figure 2*b*). However, neither *TotM* nor *TotC* knockdown was more susceptible to DTIs than their respective control genotypes (figure 2*b*). Interestingly, although the hazard ratio of the *Dif* knockdown line under DTI was more than 16 times higher than that of *TotM* knockdown under STI (46.2 ± 6.2 versus 2.8 ± 0.6), their hazard ratios were comparable after they were normalized to account for the susceptibility of their control genotypes (2.4 ± 0.4 versus 2.7 ± 0.7; figure 2*c*).

### (c) Effect of sexually transmitted infection on fecundity across RNAi strains

Sexually transmitted *Metarhizium* infections resulted in reproductive costs for female flies. Exposure to topically infected male partners initially had little impact on female reproduction, but over time, female fecundity in the infected treatment declined relative to uninfected controls (treatment × time, *F*_1,2030_ = 30.3, *p* < 0.0001; electronic supplementary material, figure S3). This pattern was consistent in all lines as there was no evidence that *TotM* or indeed any gene knockdown strain suffered greater fecundity reduction than their control genotypes (treatment × genotype, *F*_6,705_ = 1.45, *p* = 0.19). The reduction in female fecundity under STIs could not be explained by a lack of remating opportunities owing to increased mortalities of infected male partners, because male longevity did not significantly contribute to female fecundity over the course of the experiment (*F*_1,705_ = 3.5, *p* = 0.062). In addition, while the cadavers of females that had been exposed to infected males were more likely to exhibit *Metarhizium-like* fungal growth than those exposed to control males (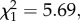 *p* = 0.017), there was no evidence that the RNAi knockdown genotypes influenced the probability of fungal growth (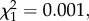 *p* = 0.97; electronic supplementary material, figure S4).

### (d) Effect of immune gene expression on survival and fecundity in uninfected flies

**Figure 3.**
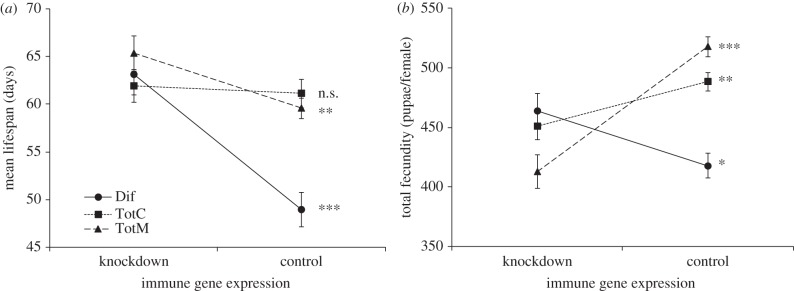
The costs of immune gene expression in the absence of infections. (*a*) Survival costs as measured by mean lifespan. (*b*) Fecundity costs as measured by total number of eclosed pupae in the first 9 days postinfection. Asterisks (*) indicate level of statistical significance (*p < 0.05; **p < 0.01; ***p < 0.001). For survival costs, statistical significance was based on Cox proportional hazard regression of the survival curves of knockdown and its combined control. For fecundity costs, statistical significance was based on one-way ANOVAs.

We found that the expression of *TotM* and *Dif*, but not *TotC*, results in survival costs for uninfected females. Both *TotM* and *Dif* knockdown flies (Act5C-Gal4/UAS-*TotM*-IR and Act5C-Gal4/UAS-*Dif*-IR), but not *TotC* knockdown flies (Act5C-Gal4/UAS-*TotC*-IR), showed enhanced survival relative to their control genotypes (*TotM*: 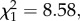 *p* = 0.0034; *Dif*: 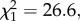 *p* < 0.0001; *TotC*: 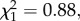 *p* = 0.35; figure 3*a*). By contrast, we found evidence for reproductive benefits of *TotM* and *TotC* expression, but reproductive costs of *Dif* expression. Both *TotM* and *TotC* knockdown females had lower total reproduction than their respective controls, whereas *Dif* knockdown females were more fecund than its control genotypes (*TotM*: *F*_1,135_ = 44.8, *p* < 0.0001; *TotC*: *F*_1,127_ = 7.6, *p* = 0.0068; *Dif*: *F*_1,129_ = 6.3, *p* = 0.014; figure 3*b*).

## 4. Discussion

Mechanisms of insect immunity are known to be pathogen-specific [37,38]. However, the extent to which insects use ecological cues to inform which responses to mount is not known. Our study shows that a gene that is upregulated in anticipation of mating provides protection against sexually transmitted *Metarhizium* infections. This finding is important because it illuminates the molecular mechanisms as well as the life-history costs and benefits which underpin immunity against STIs. In combination with previous results [15], our results imply that fruitflies demonstrate immune anticipation of mating and that immune anticipation could be a general mechanism for achieving immune specificity.

### (a) A Turandot gene that enhances immunity against sexually transmitted infections

Hundreds of *Drosophila* genes, including *TotM*, have been identified on the basis of elevated expression following immune challenges, but the functional consequences of these genes are rarely established [16–18]. This is a problem because gene expression does not necessarily translate into immunity against live pathogens [39–41]. We show that *TotM* confers protection against fungal STIs and its effects are similar in magnitude to that conferred by *Dif* to fungal DTIs.

The mechanisms through which *TotM* enhances immunity are currently unknown. All protein products encoded by the *Turandot* gene family are thought to be actively produced in the *Drosophila* fat bodies and secreted into the haemolymph, where they are hypothesized to act as protein chaperones or as signalling molecules [16,42]. Though direct tests are needed, it seems unlikely that *TotM* possesses direct antimicrobial activities similar to known antifungal AMPs, such as *Drosomycin* and *Metchnikowin*; as overexpression of another *Turandot* gene, *TotA* does not provide increased protection against Gram-negative bacterial infections [42,43]. Instead, *TotM* might help the fly to tolerate persistent fungal infections by mitigating the negative effects of the infection without actively suppressing pathogen growth [44–46]. Consistent with a role in enhancing tolerance, not resistance, we found that fungi were as likely to emerge from the control genotype flies as they were from *TotM* knockdown flies.

### (b) Mode of transmission and immunity

Fruitflies have a remarkable ability to mount immune responses which are specific to the pathogens they encounter [37,38]. Our work shows that the efficacies of their immune responses are also specific to the mode of infection transmission. STIs differ from other modes of transmission in that they tend to cause chronic low-level infections, which do not result in rapid septicaemia and increased host mortality—consequences typically associated with acute immune challenges [2]. The lower initial inoculums in our STI treatment is evidenced by the proportion of flies that exhibit fungal growth on female cadavers (5–25% for STIs and 80–95% for DTIs; VL Hunt, W Zhong, CD McClure, DT Mlynski, EML Duxbury, AK Charnley, NK Priest 2013); and the increased grooming activities we observed in the DTI treatment, which efficiently reduced pathogen load (this study; [35]). Consistent with the differences in pathogen dose between the two infection treatments, we found that sexually transmitted *Metarhizium* infections cause weak, though significant, fitness costs for females and that the expression of *TotM*, but not *Dif*, ameliorates the survival costs associated with STIs. By contrast, we found that direct topical *Metarhizium* infections cause substantial fitness costs for females and that the expression of *Dif*, but not *TotM*, helps ameliorate those survival costs. Taken together, these findings show that fruitflies have a specific mechanism for immunity against low-dose STIs and against high-dose DTIs, even for the same pathogen.

It is important to acknowledge that though we have established a role for *TotM* in immunity against low-dose STIs, we do not know whether *TotM* confers immunity against STIs *per se* or to low-dose infections more generally. We cannot dismiss the possibility that high fungal doses overwhelmed the fine-tuned protective effects provided by *TotM* or that low fungal doses masked the susceptibility of the *Dif* knockdown. Similarly, the choice of diet could confound our results, as the fecundity benefits of *TotM* and *TotC* expression might have resulted from the ad libitum access to dietary yeast in this study [47]. Another potential problem is that genetic constructs, such the Act5C driver and UAS element, may have pleiotropic effects on the life history of the fly, which could confound direct comparisons with the knockdown genotype. However, these problems are unlikely to influence our interpretations. The response to topical fungal infection in our *Dif* knockdowns was similar to that of the classic *Dif* knockout mutant [22]. Because our experiments were conducted under the same dietary conditions and because our analysis included normalizations to control genotypes, we can confidently attribute the survival reduction in *TotM* knockdown to the effect of gene expression, rather than to potential confounding factors such as diet, genetic pleiotropy or the general frailty of immune gene knockdown lines [48]. Regardless of how they confer immunity, our findings provide clear evidence that *TotM* and *Dif* are specific for different modes of fungal transmission and that their expressions have different life-history consequences for the host.

It is important to stress that we are not arguing that *M. robertsii* is predominantly transmitted sexually or claiming that it is transmitted internally during copulation. Given the proclivity of *Metarhizium* for topical transmission, we would expect there to be some non-sexual transmission, even in our STI treatments. *Drosophila* tends to aggregate on food sources, which could have increased contacts and fungal transmission in this study [49]. However, non-sexual transmission is unlikely to be substantial enough to change the interpretation of the data. First, males had been given 24 h for grooming and were subsequently placed in fresh vials, which reduced the risk of females indirectly picking up dislodged spores. Second, we found that females who mated with infected males were more likely to be infected than those that did not. And, finally, in independent experiments, infection success was substantially lower in same-sex transmission trials than in trials involving sexual transmission (21 versus 3%). Thus, although we documented that the fungus can be transmitted non-sexually, sexual transmission is primarily responsible for the observed infections in our STI treatments.

### (c) The cost of immune expression

Though many studies have documented the costs of immunity [14,47,50], the molecular and physiological basis of such costs are often poorly understood [13]. We found that under uninfected control conditions *Dif* is generally deleterious in the absence of infections. The expression of *Dif* entails both significant survival and fecundity costs, which is also supported by a previous study of *Dif* knockout mutant [48]. The costs of *Dif* expression are likely to arise from its control of AMP induction through the *Toll* pathway [22], though *Dif* might also function in other non-immunity-related processes [21]. These strong fitness costs could help to explain why *Dif* only appears to be modestly induced by direct topical fungal infections [17] and why it was not upregulated in females in response to male courtship songs (at least in their heads) [15].

By contrast, our findings for the *Turandot* genes are only partially consistent with the predicted costs of immune gene expression. We found that *TotM* has an antagonistic pleiotropic influence on the life history of the fly: though it is costly for survival, expression of *TotM* also substantially enhances female fecundity. In addition, while there was no evidence that *TotC* conferred immunity against *Metarhizium*, it did not contribute to survival cost and even enhanced female fecundity. However, unlike *Dif*, there is evidence that *TotC* and *TotM* play additional roles in reproduction. In particular, *TotC* and *TotM* are upregulated in response to exposure to male accessory gland proteins [8–12]. Perhaps *TotM* could mediate the trade-off between late-age survival and early-age reproduction, a key component of fitness in populations with fluctuating growth rates [51]. Thus, though we cannot easily tease apart the cost of expression from the additional roles played by *TotM*, the fact that its expression induces survival costs indicates that *TotM* has a long-term detrimental effect, which is an important facet of the explanation for why it is not constitutively expressed. Interestingly, *TotM* and *TotC* appear to evolve more rapidly than *Dif* [52], suggesting that they have experienced divergent or relaxed selection, perhaps as a consequence of their lower cost of expression [14,47,53].

### (d) Mating and immune anticipation in insects

Mating is frequently associated with heightened risk of contracting both ‘pure’ STIs and other opportunistic infections [3,54–56]. Such threats could be countered by upregulating immunity-related genes postmating [8–12]. However, because of the full deployment of immune responses can often take a considerable amount of time [57,58], selection is expected to favour immune anticipation of mating [7]. Though there have been few well-documented cases, immune anticipation is likely to be far more common than currently appreciated. Our study supports the hypothesis that female fruitflies can mitigate the risk of contracting sexually transmitted fungal infections during mating by pre-emptively upregulating *TotM* [15]. More generally, there are many other biological scenarios associated with elevated disease risk for which we would expect immune anticipation to be advantageous, such as feeding (as has been documented in bed bugs; MT Siva-Jothy, E Harney, W Zhong 2013, unpublished data) and crowding of conspecifics [59–61]. A particularly tantalizing possibility is that the control of many immune genes including *TotM* [62] by circadian clock genes might reflect ‘anticipation’ of predictable fluctuations of disease risk over the course of 24 h. Thus, the courtship-induced, pre-emptive upregulation of *TotM* might be representative of a general pattern of immune anticipation in insects, underlining the intimate link between brain, behaviour and immunity [63,64].

## Data accessibility

All supporting data are accessible in the electronic supplementary material.
